# Moving towards a continuum of safer supply options for people who use drugs: A qualitative study exploring national perspectives on safer supply among professional stakeholders in Canada

**DOI:** 10.1186/s13011-022-00494-y

**Published:** 2022-10-08

**Authors:** Annie Foreman-Mackey, Bernie Pauly, Andrew Ivsins, Karen Urbanoski, Manal Mansoor, Geoff Bardwell

**Affiliations:** 1 British Columbia Centre on Substance Use, 400-1045 Howe Street, V6Z 2A9 Vancouver, BC Canada; 2 Department of Medicine, University of British Columbia, St. Paul’s Hospital, 608-1081, V6Z 1Y6 Burrard Street, Vancouver, BC Canada; 3 Canadian Institute on Substance Use Research, University of Victoria, 2300 McKenzie Avenue, V8P 5C2 Victoria, BC Canada; 4 School of Nursing, University of Victoria, Box 1700 STN CSC, V8W 2Y2 Victoria, BC Canada; 5 School of Public Health and Social Policy, University of Victoria, 3800 Finnerty Road, V8P 5C2 Victoria, BC Canada; 6 School of Public Health Sciences, University of Waterloo, 200 University Ave. West, N2L 3G1 Waterloo, ON Canada; 7 British Columbia Centre on Substance Use, 400-1045 Howe Street, V6Z 1Y6 Vancouver, BC Canada

**Keywords:** Safer supply, Overdose prevention, Stakeholder perspectives, Canada, Qualitative research

## Abstract

**Background:**

Novel public health interventions are needed to address the toxic drug supply and meet the needs of people who use drugs amidst the overdose crisis. Safer supply – low-barrier distribution of pharmaceutical grade substances – has been implemented in some jurisdictions to provide safer alternatives to the unregulated drug supply, yet no studies to date have explored professional stakeholder perspectives on this approach.

**Methods:**

We used purposive sampling to recruit professional stakeholders (n = 17) from four locations in British Columbia, Ontario, and Nova Scotia, including program managers, executive directors, political and health authority representatives, and healthcare providers involved in the design, implementation, and/or operation of safer supply programs in their communities. Semi-structured, one-to-one interviews were conducted, and interview data were coded and analyzed using thematic analyses.

**Results:**

Participants defined safer supply as low-barrier access to substances of known quality and quantity, offered on a continuum from prescribed to a legal, regulated supply, and focused on upholding autonomy and liberation of people who use drugs. Stakeholders expressed support for safer supply but explained that current iterations do not meet the needs of all people who use drugs and that implementation is limited by a lack of willing prescribers, stigma towards people who use drugs, and precarity of harm reduction programs to political ideology. Stakeholders expressed strong support for wider-reaching approaches such as decriminalization, legalization, and regulation of substances as a way to fully realize a continuum of safer supply, directly address the overdose crisis and toxic drug supply, and ensure equity of access nationally.

**Conclusion:**

The results of this study highlight the need for innovative strategies to address the overdose crisis and that safer supply has the potential to benefit certain people who use drugs. A one-size-fits-all approach is not sufficient and the perspectives of professional stakeholders should be considered alongside those of people who use drugs when designing and implementing future safer supply.

## Background

Canada is in the midst of a public health emergency, with 24,626 opioid toxicity deaths reported between January 2016 and June 2021, accounting for 19.1 deaths per 100,000 individuals [1]. Contamination and toxicity of the unregulated drug supply is fueling the overdose epidemic, with 87% of accidental apparent opioid toxicity deaths involving fentanyl [1], and increasing detection of benzodiazepines such as etizolam and other psychoactive compounds such as xylazine [2, 3]. The crisis has widespread consequences, including being one of the leading causes of accidental death [4] and contributing to a decline in life expectancy [5, 6].

A number of public health and harm reduction interventions have been implemented across Canada to curb the overdose crisis, including supervised consumption services (SCS), naloxone distribution programs, drug checking technologies, and expansion of oral and injectable opioid agonist therapy (OAT/iOAT) [7]. It is estimated that in the Canadian province of British Columbia (BC) alone, the combined impact of harm reduction interventions implemented and available in the province between April 2016 and December 2017 prevented 3000 overdose deaths during this same time period [8]. Scale-up of SCS was also shown to bring about other related health benefits by addressing ongoing unmet needs [9]. Despite these steps taken, rates of fatal and non-fatal overdose continue to rise in tandem with the drug supply toxicity [10], exacerbated further by the COVID-19 pandemic [11], and services are falling short of meeting the needs of all people who use drugs [12].

Some jurisdictions have experimented with “safer supply” – low-barrier distribution of pharmaceutical grade substances – to directly address the toxicity of the drug supply and engage with people who use drugs for whom conventional treatment services are not sufficiently suitable or desirable. The Canadian Association of People who use drugs (CAPUD) identifies safer supply as “*legal and regulated supply of drugs with mind/body altering properties that traditionally have been accessible only through the illicit drug market*” and includes a range of opioids, stimulants, and hallucinogens [13]. CAPUD explicitly outlines that opioid substitution treatments, such as methadone, buprenorphine, and slow release oral morphine should not be considered safer supply given that they do not bring about the same mind and body altering properties that people who use drugs seek in recreational substances [13].

Though the concept safer supply is novel, the provision of pharmaceutical grade opioid alternatives to illegal substances is not new. Existing OAT programs range from methadone and buprenorphine [14] to injectable diacetylmorphine and hydromorphone [15], and more recently to include low-barrier tablet hydromorphone [16], slow release oral morphine [17], and transdermal fentanyl [18]. Though OAT programs have demonstrated effectiveness in treating opioid use disorder [15, 19] and reducing all-cause mortality among people with opioid use disorder [20], certain people who use drugs are not being reached nor sufficiently accommodated, including people who do not inject substances, those in rural and remote communities, and individuals for whom routine engagement with the healthcare system and traditional treatment approaches are not feasible [12, 21].

Canada’s response to the overdose emergency will continue to be limited if we rely uniquely on traditional treatment models; novel harm reduction approaches such as safer supply are needed to expand reach and diversify the toolkit of interventions available to meet the needs of people who use drugs. Some physicians have also opted to prescribe hydromorphone tablets off-label to individuals at high risk of overdose [22, 23] and smaller-scale safer opioid supply distribution programs have also been implemented in the form of low-barrier hydromorphone distribution programs via a biometrics-storage locker since 2019 [12, 24]. Though establishment of safer supply programs like these is a necessary step forward, many are in a pilot program format, operate at small capacity, and continue to be under-evaluated [25]. BC more recently introduced a public health order that permits nurses to prescribe some controlled substances [26], and the BC Centre on Substance Use released “Risk Mitigation Guidelines” in 2020 that outline prescription guidelines to provide pharmaceutical-grade substances to those: at risk of COVID-19 infection or with confirmed or suspected cases of COVID-19; individuals who have a history of ongoing active substance use; and those deemed at high risk of withdrawal, overdose, craving, or other harms related to substance use [27].

Implementation and effectiveness of harm reduction services have been shown to be contingent on local, organizational, and health systems level contextual factors and shaped by broader social, political, economic, and physical structures underpinning health and health disparities [28, 29]. It is therefore critical to explore and understand the conditions in which novel health interventions, such as safer supply, are designed and implemented. To ensure successful implementation and achievement of intended outcomes, input from both people who use drugs and other professional stakeholders is essential to inform and adapt health interventions to the unique needs of communities.

To date, minimal literature has directly explored professional stakeholder perspectives on safer supply approaches [30] or the varied ways in which safer supply is conceptualized both in design and implementation. Therefore, we undertook this study to explore stakeholder perspectives on key features of safer supply, support for and reservations about this approach, facilitators and barriers to implementation, and visions of ideal safer supply interventions.

## Methods

This qualitative study was undertaken as part of an independent evaluation of a novel safer supply pilot program offering low-barrier access to hydromorphone tablets via a biometric dispensing machine [31, 32]. Interviews were conducted with professional stakeholders involved in the design, proposal, implementation, and/or operation of the program (n = 17) to elicit perspectives on safer supply broadly as well as specific facilitators and barriers faced in the implementation of safer supply in their jurisdiction. Participants were purposively recruited by the program lead at each of the four proposed biometric opioid dispensing machine pilot program locations in Canada – Victoria, British Columbia (n = 4), Vancouver, BC (n = 6), London, Ontario (n = 2), and Dartmouth, Nova Scotia (n = 5) – and selected based on their expertise and involvement in the implementation and operation of safer supply programming in their communities. Three of these locations were in the pre-implementation stage while one location was operating the program at the time of data collection. Stakeholder roles included: program managers and executive directors (n = 7), political and health authority representatives (n = 3), and healthcare providers: physicians (n = 5), nurse (n = 1) and pharmacist (n = 1).

Between June and September 2021, the lead author conducted one-to-one, semi-structured, in-depth interviews with stakeholders over the phone or Zoom. An interview guide was used to facilitate the exploration of a range of topics, including: local context with respect to overdose risk; availability of overdose interventions; perspectives on safer supply and vision for the future; facilitators and barriers to implementation and scale up; experiences implementing safer supply; and recommendations for program operation. In this article, we focus on perspectives on safer supply and facilitators and barriers to implementation in general, while subsequent articles will explore intervention-specific topics. Participants provided written informed consent and were assigned an individual number to ensure anonymity. Interviews lasted between 15 and 110 min and were audio recorded and professionally transcribed. Data collection ended once no other potential participants identified by the program leads expressed interest in participating in the study. Given the small size of the pilot, in order to protect participant identities, we do not disclose participant locations after each quotation or specific demographic identifiers. While interviewees participated as professional stakeholders, some also identified as people with lived/living experience of drug use.

Interview data were imported and coded using NVivo Qualitative Data Analysis Software (version 12). Applied thematic analysis [33] was conducted primarily by the lead author, with identified categories and findings discussed with and validated by the senior author throughout the analysis process. Full transcripts were first reviewed using a line-by-line deductive approach to identify text relevant to *a priori* categories as outlined in the interview guide, followed by an iterative, inductive approach to capture emerging themes (e.g. future of safer supply) and subthemes (e.g. importance of people who use drugs leading program development and operation) [34]. Preliminary findings were also reviewed by co-authors.

This study was approved by the University of British Columbia/Providence Health Care Research Ethics Board.

## Results

Stakeholders provided insight into the way that they conceptualize and define safer supply, their varied perspectives regarding support for and reservations about safer supply, facilitators and barriers to implementation, and vision for the future of safer supply.

### Defining “Safer Supply”

Participants acknowledged the variety of ways that safer supply is conceptualized and defined. However, they identified several common features of what they viewed as “safer supply”: low barrier access to alternatives to the toxic illegal drug supply that are the exact substance or as close to comparable as possible, of known quantity and quality, and in the formulation needed for individuals’ desired method of consumption. Stakeholders characterized the ideal safer supply approach as a continuum of safer alternatives ranging from prescription-based to a legal, regulated supply able to meet the diverse needs of all people who use drugs. Participants expressed that substances should be accessible for a range of reasons, including euphoria and as a means to cope with pain and trauma, and aimed at supporting the liberation and agency of choice of people who use drugs. Using this definition, stakeholders felt that safer supply does not yet exist in Canada.

*Right now I think we’ve got some close approximations* [of safer supply]*… working around the edges with what’s possible… I think safe supply raises everyone’s expectations and… it’s not realistic. It’s not what we have. I mean we want that but we need to be getting real about how we do it then and acknowledging that we don’t have it and there’s no clear path to get it at this point in time.* (P6 – health authority)

Despite the shortcomings of existing safer supply programs, participants expressed broad support for safer supply and strong views about what characteristics form the foundation of a safer supply approach, as outlined below.

#### Substances and accessibility

Stakeholders reported that the effectiveness of safer supply programming was contingent on how close the substances on offer were to an individual’s drug of choice and how accessible the services were to diverse people who use drugs. Comparable substances address some need, but “*we see gradations in the effectiveness of safe supply depending on how close to the actual drug we’re getting*” (P1 – program lead). As such, the majority of participants explained that the substances available through safer supply should be diversified to include not only opioids, but also stimulants, and in the formulation that individuals need.

*An ideal program that could help not just people who use opioids, but people who use stimulants as well, and other substances… and I think it has to make the local – like we can’t just base one safe supply program for all of Canada, because every location’s different…it’s being flexible and adaptable… and diversifying the molecules that are out.* (P5 – program lead)

Stakeholders also explained that safer supply approaches should be low-barrier, flexible, and have broad eligibility requirements. Individuals should be permitted to carry doses as required and avoid mandatory witnessed dosing. One healthcare provider explained:

*A lot of our patients don’t have stable housing. Their phones work intermittently. We need to be flexible in terms of hours and like when they can just show up and see a doctor because at my other clinic, even though most of my patients are stable, the ones that are really struggling with having a phone that works so I can reach them to call them in for a urine drug screen or remind them of an appointment. But like I think for the safe supply clientele it’s even more vital to have a really flexible program*. (P3 – healthcare provider)

Participants acknowledged that people use substances for a range of reasons, including euphoria, and underscored that individual reasons to use substances should not preclude their ability to access safer supply programs. One participant explained that “*we’re looking to help people, not necessarily change their substance use… to stabilize so that they can live with dignity and hope*” (P15 – program lead). Participants felt that safer supply would be most effective if tailored to the unique patterns of substance use within their community as well as the unique needs of the individual accessing this service.

#### Continuum of safer supply models that uphold autonomy and liberation of people who use drugs

Stakeholders believed that safer supply is not a “one-size-fits-all” approach and that implementing a range of safer supply options acts to liberate people who use drugs, bringing agency of choice and autonomy, and respecting the fact that people who use drugs are “*the experts of their own lives*” (P13 – program lead). Participants explained that “*Choices are really motivating factors, especially in something as complex as addiction. So anything that gives more choice and more options in unabashedly progress*” (S8 – program manager), and “*People need options, and there’s no other type of health concern or health issue that you could be offered like two different medications and that’s it*” (P5 – program lead). In addition, participants felt that safer supply ideally is:

*an extension of human rights… people have the right to have fun… also the right to relieve pain and to cope with trauma and violence in the ways that make sense to them… I just believe that people have autonomy over their bodies and should be able to make decisions about what they put in their bodies.* (P14 – program manager)

As such, a diversity of models and interventions are needed to meet the needs of people who use drugs and flexible enough to find the right combination of approaches for each individual at that point in time: “*it’s never just one solution and we need a variety of options for a variety of people*” (S14 – program manager) and *“there’s no one model that’s going to be the panacea of safe supply”* (P16 – healthcare provider).

[Safer supply] *centres on patient need and choice, and patient goals. So treating each as an individual, right? And so what is the desired drug that they want for safe supply? What is the desired route? How can we get them the correct volume? What is actually their goal around, you know, street level drug use? Is it to, you know, be completely abstinent from street level drugs? Is it to reduce to a certain level that they feel safer and that they can afford more readily? So, you know, the ideal safe supply, I think, centres the patient, and does not try to use a blanket one-size-fits-all kind of guideline type of approach.* (P17 – healthcare provider)

Participants pointed to the need for a continuum of safer supply strategies from high-barrier, medicalized opioid agonist therapy to low-barrier, compassion club models or dispensaries, with people who use drugs “*involved in all levels of leadership… implementation and evaluation to actually delivering services and supports”* (P14 – program manager). Stakeholders explained that initiatives will not succeed in achieving their intended outcomes if people who use drugs are not leading the process, and that the best model of safer supply is “*one that is uniquely fitted into each community… ask people what they need, do that, and then keep asking them what’s not working and keep changing to meet their needs”* (P16 – healthcare provider).

Although it was generally acknowledged that it is easiest to introduce interventions by way of pilot programs within a medical system, most stakeholders felt that it was problematic to have physicians as “gatekeepers” to safer supply given that access will be contingent on finding prescribers willing to participate. While a medicalized pilot program can act as a proof of concept and steppingstone, stakeholders felt that *“there is zero chance that the medical model can meet the needs of everybody… I just cannot see a version of the world where there’s enough practitioners to prescribe this”* (P17 – healthcare provider). That being said, some participants explained that if safer supply were to remain in the medical domain, then it should be approached as a public health intervention – like a vaccine or naloxone program – rather than a medical intervention: “*you just were screened and fell into category A which means you’re eligible for option A, and then you just get that”* (P16 – healthcare provider).

### Stakeholder perspectives on safer supply

#### Support and facilitators

Stakeholders were unanimous in their broad support for safer supply given that existing approaches to address overdose are not sufficient and that there is an urgent need to find creative solutions to keep people alive in the midst of the ongoing overdose epidemic: “*we’ll try anything at this point*” (P6 – health authority). Participants acknowledged the benefit of providing safety and stability for people living chaotic lives: “*it allows that person to slow down and not spend their entire day trying to secure the funds or the drugs… just to make it so they don’t feel dope-sick all day long”* (P3 – healthcare provider).

*I think even semi-effective safe supply takes some of the urgency out of someone’s immediate need and allows people to make safer more informed decisions about what they do purchase when it’s not in a crisis situation… gives some relief and reduces the urgency…* [especially for] *folks who just wanted to stay safe but… were not in a current position to want to reduce their use of street opiates.* (P1 – program lead)

Despite discrepancies between safer supply quantities able to be prescribed by physicians and individual tolerance levels, some stakeholders felt that there was still benefit: “*Even if what they’re getting is not enough it means that they’re most likely reducing the street substance use. It means more likely they’re able to be less sick that day… it’s better than nothing”* (P13 – program lead). Other healthcare providers described improvements in both medical and non-medical outcomes among individuals accessing safer supply:

*We get feedback from the community. One of the best ones is when the street level cops no longer recognize folks because they’ve put on weight and they’re looking so healthy… some of the most profound gains* [from safer supply] *are going to be in those social service metrics.* (P17 – healthcare provider)

Physician participants explained that having a community of prescribers rather than individuals acting in isolation helped them feel more comfortable prescribing safer supply, especially given the expressed fear of audit and lack of explicit guidance from the regulatory Colleges: “*a team of physicians who were able to support each other in taking some more progressive steps with their prescribing practices… I think a lone physician would struggle quite a bit more taking some of those perceived risks”* (P1 – program lead). They felt that communities with established groups of willing prescribers could more readily implement more progressive health interventions, such as safer supply.

Safer supply was also discussed as providing opportunities to build relationships with people who may not otherwise engage with the health system, and as a way to protect individuals from harm and suffering for whom intensive, regimented medical treatment is not realistic or desired. For many people who use drugs, interventions such as opioid agonist therapy “*is not the treatment that they need or they want…* [it is] *really restrictive and so it doesn’t meet the needs of a lot of people*” (P5 – program lead).

Participants explained that implementation of safer supply programs and other harm reduction services is facilitated by the presence of organizations comprised of people who use drugs and a range of trusted, strong existing connections in their community. One stakeholder explained:

*We wouldn’t be anywhere as close to where we are if it wasn’t for the leadership of* [people who use drugs]… *that’s the biggest thing and then their national contacts and knowledge… the rest of us have kind of played a peripheral role. Their leadership has been the biggest facilitating factor*. (P7 – healthcare provider)

Communities in which groundwork had already been done to gain widespread acceptance of harm reduction could more easily expand existing interventions to include safer supply. For example, implementation and scale-up of diverse harm reduction interventions in Vancouver, BC, has been possible due to decades of community-led work and activism by groups such as the Vancouver Area Network of Drug Users [35, 36]. One stakeholder explained that: “*The fact that we’re quite an open, liberal, forward thinking city definitely makes it a lot easier for programs like we have and I think that if you were in a conservative city that it would be a lot tougher*” (P11 – healthcare provider). Stakeholders highlighted the multitude of ways that local contextual factors shape program implementation.

#### Reservations and barriers

Stakeholders had some reservations about safer supply and cautioned that safer supply is *“not the panacea… it’s one of the solutions*” (P15 – program lead). They expressed concern that current iterations of safer supply are only “half measures”: “*it’s not enough and it’s not actually listening to what folks need*” (P13 – program lead). Stakeholders from BC felt that the Risk Mitigation Guidelines implemented in BC in the COVID-19 context are at risk of being rolled back as the pandemic subsides. Stakeholders were also worried that safer supply in its current form may turn into another “treatment model” that overmedicalizes substance use and fails to meet the unique needs of some people who use drugs.

Physician participants described the internal struggle they felt when prescribing safer supply given their medical training and questions about whether they are doing more harm than good. For example:

*My reservations come mostly from my medical training and awareness of the potential harm of these drugs and even though I support it and I’m learning how to do it and committed to figuring that out, the idea that we’re prescribing these substances not really knowing what people are going to be doing with them and is it possible that we’re creating some harm with our prescriptions which is really hard to swallow and challenging and for some of the limited safe supply that we’ve been doing creates a lot of worry and anxiety as a prescriber so trying to balance the known harms of not prescribing safe supply with the unknown harms of prescribing it is really tricky and then trying to weight those potential benefits and harms from a public health perspective versus my own pen and prescription pad is really tricky.* (P7 – healthcare provider)

Some prescribers were mixed about whether safer supply is achieving its intended outcomes: “*I’ve run around 200 people through the ring around this and maybe I’ve got like five people that it benefits. The rest I don’t have any real solid indicators that it’s helped”* (P12 – healthcare provider). Other physicians spoke about having their hands tied to prescribing guidelines and knowingly having to prescribe doses of pharmaceuticals that are insufficient to meet individual levels of tolerance: “*I’m afraid that we’re always chasing the tail of the street supply to keep up with tolerances and changing additives”* (P17 – healthcare provider).

Physicians and pharmacists were also fearful about liability and being audited by their regulatory Colleges: “*All of us are in fear of our licenses. All of us are in fear of College complaints”* (P12 – healthcare provider). Participants described ideological differences between medical models of care and harm reduction models that created barriers to adopting safer supply into their practices.

[Physicians have] *been told for almost a decade that they’re prescribing too many opioids, and now we’re saying please prescribe opioids. So I think that’s legitimate… the non-early adopters are waiting for published evidence… Also getting the medical establishment to wrap their heads around euphoria as a goal of treatment or support is going to take some time too. Doctors don’t like people to get high… so it’s kind of the long history of being told not to prescribe opioids, as well as a bit of a wait and see for evidence. But you also need to have enough people to gather evidence… it’s a bit of a Catch-22.* (P17 – healthcare provider)

Given the lack of explicit guidance from many of the regulatory Colleges and hesitation among prescribers, stakeholders identified the lack of willing prescribers as a critical barrier to scale up of safer supply. They felt that continuing to have physicians as the “gatekeepers” to safer supply creates a “*serious bottleneck”* (P12 – healthcare provider).

In addition, Canada is a large country, and stakeholders described huge disparities in access to harm reduction services between and within provinces and territories due to ongoing stigma towards people who use drugs, as well as political will and prevailing political ideology: “*it’s up to the elected governments in each of those jurisdictions to make the calls* [about harm reduction] *and this is actually just a human rights issue. It’s frustrating that political decisions are made that are so life and death for people”* (P14 – program lead). Participants spoke of the precariousness of harm reduction programming to changes in political ideology and the need to move beyond pilot programs towards more sustainable interventions.

*There’s always the precarity of harm reduction and the fact that when all we do is implement pilot studies and the pendulum swings on the other side of the political continuum and all the sudden, we’re stuck with a conservative government, all of that gain that we made could simply disappear before our eyes. And so, that’s why we need to stop doing pilot studies and studying these issues to death, studying these people to death, and actually roll out more robust, long-term funding for projects.* (P16 – healthcare provider)

Participants expressed an urgent need to implement safer supply and other harm reduction interventions in a way that ensures sustainability and protection against roll-back if there is a change in political leadership.

#### The future of safer supply in Canada

Stakeholders underscored the importance of being innovative in the approach to safer supply as a means to protect people who use drugs from harm, and expressed a vision for the future of safer supply that extends beyond the reach of current programming towards larger-scale, systemic change. Participants called for greater continuity of access to safer supply and a coordinated, national rollout to minimize disparities between and within provinces and territories.

*I would hope that people can receive the same health care here, or access to safe supply. If I had to move, because my family was moving, I should be able to access that somewhere else. That’s a huge barrier, that there’s no continuity across the country… my hope is that we could have like a… national rollout of safe supply. Or even it be adopted just by family doc*[tors] *in general.* (P5 – program lead)

Other stakeholders urged for safer supply to become standard practice for physicians – as another tool in their toolbox of services they are able to offer to patients – and for the Colleges of Physicians to establish some basic guidelines and standards.

*I don’t think safe supply necessarily has to be a program, right? I would like it to just be a tool in any general practitioner’s, whether it’s family medicine or even like general internal medicine, that they use alongside diabetes care, right? It should just be another tool in your toolbox to care for an individual. It doesn’t need to be labeled as this special program for special people, right?* (P17 – healthcare provider)

Participants pointed to larger systemic factors shaping substance use and drug-related harm, such as prohibition and failed drug policies. They felt that to fully realize a continuum of safer supply and protect the health and dignity of people who use drugs, Canada must ultimately move towards decriminalization and full legalization and regulation of substances to address the root causes of the overdose and drug poisoning crisis: “*We can scale up* [existing harm reduction programs] *but then what we’re doing is we’re providing Band-Aid solutions to systemic problems*” (P15 – program lead). Other participants emphasized how safer supply is tied to the push for decriminalization. For example:

*Decriminalization, that’s a really important component of this. To have a regulated safe supply, we can’t have them be illegal… I think the conversation for decriminalization will continue to move along and I think that regulated safe supply will probably have to follow that, so I don’t think you can do one without the other. I think you can decriminalize without a safe supply; I don’t think you can create a safe supply without decriminalization.* (P9 – program lead)

Participants expressed strong support for wider-reaching approaches like decriminalization, legalization, and regulation of substances as a way to directly address the overdose crisis and toxic drug supply, and to ensure equity of access within and across jurisdictions.

## Discussion

This study explored the perspectives of professional stakeholders on safer supply design and implementation in three provinces and across four cities, of which three were in pre-implementation and one had an operational safer supply biometric dispensing machine program. They identified several key features of safer supply programming, including: providing substances that are comparable to that of the individual’s drug of choice, low-barrier and flexible in design, bringing agency of choice and autonomy to people who use drugs, and offering a range of formats, from prescriber-based therapy to compassion club models or dispensaries. Given that existing interventions are not sufficient to meet the needs of all people who use drugs, stakeholders were broadly supportive of safer supply as a way to urgently address the overdose crisis, to save lives, and to foster relationships with people who use drugs who are most at risk of overdose and who may not otherwise access health services. However, they felt that implementation was limited by prescriber hesitancy and fear of audit given lack of explicit guidance from regulatory Colleges, as well as the expansiveness of Canada with continued disparities in access due to stigma and precarity of harm reduction programs to political ideology. Participants called for safer supply strategies that extend beyond a prescriber-based model towards a multiplicity and diversity of safer supply approaches able to meet the needs of all people who use drugs.

Many studies have described the leadership of people who use drugs in implementing harm reduction services in their communities [e.g. 29–32] and the importance of including people who use drugs in the design, implementation, operation, and evaluation of harm reduction services [e.g. 33–37]. Though the perspectives of people who use drugs were not explored in this article, this is a necessary and critical next step and researchers have started to publish these perspectives [39, 40]. However, inclusion of the perspectives of broad professional stakeholders – as explored in this study – is also essential to ensure that roll-out of harm reduction approaches are contextually-appropriate, effective, and sustainable. Though this is the first study – to our knowledge – to examine stakeholder perspectives on safer supply, prior literature outlines stakeholder perspectives on other harm reduction interventions, such as SCS [e.g. 38–42] and fentanyl drug checking [53]. These studies highlight the importance of including diverse community perspectives in the design and implementation of programs and services.

The Health Equity Implementation Framework (HEIF) underscores that interventions must be adapted to unique contexts and address determinants of health disparities to ensure that they promote health equity and support the health of marginalized populations [29]. To do this effectively, a deeper understanding is needed of macro-level (e.g. restrictive drug policies, stigma towards people who use drugs, criminalization), meso-level (e.g. public health, government, health institutions), and local-level factors (e.g. substance use patterns, population demographics, rural or urban contexts, local unregulated supply dynamics and composition) to inform the development and implementation of harm reduction services such as safer supply.

Stakeholders pointed to facilitators and barriers to implementation at each level outlined in the HEIF. At the macro-level, sociopolitical factors such as prohibition, stigma, and criminalization of people who use drugs have fueled the current overdose crisis and toxic unregulated drug supply and limited the establishment and scale up of services for people who use drugs [24]. To this end, participants called for decriminalization as well as moving towards legalization and regulation of substances to address these root, systemic issues [31, 32, 54]. In addition, the COVID-19 pandemic exacerbated risk of harm among people who use drugs and impeded access to harm reduction services due to public health restrictions [11, 24, 31].

At the meso-level, lack of explicit guidance from regulatory Colleges along with restrictive prescribing guidelines caused early iterations of safer supply to be inadequate in meeting the needs of all people who use drugs, in particular given the discrepancy in the amount prescribed compared to tolerance levels. An ethics review commissioned by the BC Ministry of Mental Health and Addictions asserts that the benefits of providing pharmaceutical alternatives to people who use drugs in the context of high rates of overdose deaths outweighs the harms, and that failure to prescribe would be a violation of prescribers’ fiduciary obligation to act in the best interest of their patients [55]. Participants from our study also pointed to the precarity of harm reduction interventions to shifts in the prevailing political ideology – particularly at the provincial level (beyond the Colleges) – as another implementation barrier. They expressed concern that existing safer supply programs may be scaled back if there is a shift in political leadership, as seen in Alberta and Ontario when access to SCS was significantly scaled back after newly elected conservative provincial governments came to power [56, 57]. For example, election of a conservative Ontario provincial government in 2018 brought about a new process for SCS application approval that included more restrictive requirements and a cap on the number of sites that the government could approve, leading to closure and loss of funding for some existing sites, and creating insurmountable administrative burden for many smaller communities with fewer resources [56]. Stakeholders also called for coordinated roll-out across the country to ensure equity and continuity of access, and to minimize disparities in access to harm reduction services between jurisdictions.

At the local level, participants in this study echoed the importance of being attentive to and able to adapt to unique local contexts in the design and implementation of safer supply approaches. Having strong community connections and collaboration with organizations comprised of people who use drugs facilitated implementation of safer supply and other harm reduction services. Some prescribers expressed hesitation and felt mixed about whether safer supply is achieving its intended outcomes, as described elsewhere [e.g. 50–52]. Many stakeholders also called for the expansion and diversification of the substances offered through safer supply to more adequately meet the needs of people who use stimulants and people who smoke drugs, yet no programs to date have been tailored to directly address these unique patterns of use and methods of consumption [58, 59].

Finally, participants urged that the future of safer supply should include a multiplicity and diversity of safer supply approaches to address the shortcomings of a “one-size-fits-all” approach and better meet the needs of people who use drugs, ranging from medicalized models to dispensary, compassion club type models that operate outside of a prescriber-based model. A broader vision of safer supply and placing a particular focus on implementing lower-barrier, flexible approaches to safer supply has the potential to extend reach of these programs to people who are not accessing services, and to address limitations of existing approaches, such as the scalability of highly medicalized OAT models [12, 32] and restrictions on take home doses for individuals enrolled in injectable OAT programs [60].

This study has several limitations. First, participants were recruited from four locations across Canada and may not be representative of stakeholders in all jurisdictions, especially in cities where safer supply is not available in any form. In addition, participants were identified by program leads due to their involvement in the implementation of a safer supply pilot project. Therefore their baseline level of support for safer supply may be greater than that of other stakeholders and the broader public. Further research is also needed to explore the perspectives of health care providers not involved in safer supply programs to understand barriers for those who may be hesitant or unwilling to prescribe safer supply. Though the sample size of this study was small in total number, there was repetition in participant responses in later interviews, indicating that saturation had been reached on the key topics of interest [61]. Future research, including community-based, ethnographic, and mixed methods approaches, should investigate perspectives and experiences of people who use drugs accessing safer supply to gather a complete understanding of stakeholder perspectives.

## Conclusion

This qualitative study highlights the need for innovative strategies to address the overdose crisis and that safer supply has the potential to benefit certain people who use drugs. Stakeholders maintained that a one-size-fits-all approach is not sufficient to meet the needs of diverse people who use drugs, and that roll out must be adapted to the local context and individual needs while also addressing larger contextual drivers including regulatory policies and criminalization. Considering the perspectives of professional stakeholders alongside those of people who use drugs is critical when designing and implementing future safer supply strategies to address the overdose crisis. Given the urgency of the overdose crisis, toxicity of the unregulated drug supply, and concurrent COVID-19 pandemic, the findings of this study can be used to inform policy and practice regarding safer supply implementation across Canada.

## Data Availability

The qualitative datasets for this study are not publicly available given the sensitive nature of the topic, including confidential information that could compromise participant confidentiality and consent.

## List of abbreviations

BC: British Columbia.
CAPUD: Canadian Association of People who Use Drugs.
COVID-19: Coronavirus Disease 2019.
HEIF: Health Equity Implementation Framework.
iOAT: Injectable opioid agonist therapy.
OAT: Opioid agonist therapy.
SCS: Supervised consumption services.
